# Gender-specific responses to multifaceted factors associated with disordered eating among adolescents of 7th to 9th grade

**DOI:** 10.1186/s40337-021-00524-3

**Published:** 2022-01-10

**Authors:** Duan-Rung Chen, Grace Sun, Brianna Levin

**Affiliations:** 1 Institute of Health Behaviors and Community Sciences, College of Public Health, National Taiwan University, No. 17, Xu-Zhou Road, Taipei, 100 Taiwan; 2 University of Southern California, Los Angeles, CA USA; 3 Global Health Program, College of Public Health, National Taiwan University, Taipei, Taiwan

**Keywords:** Disordered eating, Gender difference, School environment, Family, Adolescents

## Abstract

**Background:**

The prevalence of disordered eating is increasing among adolescents in Asia. The prevalence and predictors of disordered eating in boys have often gone unrecognized. This study examined gender-specific responses to multifaceted factors associated with disordered eating, including personal, behavioral, family, and school-related characteristics.

**Methods:**

After excluding responses with incomplete information, a sample of 729 adolescents (48.97% boys) between the ages of 13 and 16 were surveyed through convenience sampling from 37 classrooms in three junior high schools in New Taipei City of Taiwan were analyzed. The Eating Attitudes Test-26 questionnaire was used to identify disordered eating.

**Results:**

No difference in the prevalence of disordered eating between the genders was found. Adolescent girls exhibit a preoccupation with fatness and a desire to be thinner, whereas boys are more likely to engage in extreme dieting behaviors such as vomiting, keeping the stomach empty, and avoiding sweets. Girls engaging in disordered eating reported relatively high levels of interpersonal stress involving family member weight-teasing, low peer acceptance, and high peer pressure to control weight. High intensity of regular exercise was found in girls with disordered eating. The perception of body weight is a more critical factor of engaging in disordered eating for boys than girls. Adolescents with immigrant parents were associated with disordered eating among both genders.

**Conclusions:**

Changing gender-specific weight-related norms in schools and families is essential to reduce the prevalence of disordered eating, particularly among girls. Future studies using representative samples to confirm this study’s findings are warranted.

**Supplementary Information:**

The online version contains supplementary material available at 10.1186/s40337-021-00524-3.

## Plain English Summary

This study examined gender-specific responses to multifaceted factors associated with disordered eating, including personal, behavioral, family, and school-related characteristics. This study did not discover a significant difference in the prevalence rate of disordered eating between the genders. However, adolescent girls appeared preoccupied with fatness and a desire to be thinner. Boys tended to engage in extreme dieting behaviors such as vomiting after meals, keeping their stomach empty, and avoiding sweets. Relative to adolescent boys, adolescent girls who engaged in disordered eating reported more interpersonal stress from family members’ weight-related teasing, friend pressure to control their weight, low peer acceptance, and being in an extended family. A high frequency of regular exercise was found in girls engaging in disordered eating. The self-perception of body weight seems to be a more critical factor for boys than girls. Boys with fathers of high education and boys who perceived more peer support tended to engage in disordered eating. More attention should be directed to exploring male-specific factors related to disordered eating. The background of immigration is associated with disordered eating among both genders. As the number of mixed-culture marriages increases in Taiwan, this finding highlights the need to explore the health of immigrant parents' children. This study’s strengths and limitations must be considered in interpreting the results. The study’s key strength is its assessment of various personal, behavioral, family, and school environmental variables of potential relevance to disordered eating among the genders in Taiwan. However, the measures adopted in the current study were brief and based on self-reporting, suggesting a need for further exploration of gender differences in disordered eating in a more extensive and comprehensive population-based study.

**Supplementary Information:**

The online version contains supplementary material available at 10.1186/s40337-021-00524-3.

## Background

Disordered eating comprises a continuum of irregular eating attitudes and behaviors ranging from excessive weight concern and body shape dissatisfaction to extreme weight control methods and binge eating [1]. Disordered eating has been theorized to be linked to the diet cycle, in which restricted eating causes physical and psychological deprivation, which leads to binge eating, leading to mental health effects such as low self-esteem that can contribute to body dissatisfaction; this dissatisfaction then results in further restricted eating, and the diet cycle is thereby repeated [2].

Frequent dieting, compulsive eating habits, and chronic weight fluctuations are common characteristics of problematic eating behavior that may progress to an eating disorder if left untreated. Extreme dieting that precedes binge eating is among the most crucial predictors of eating disorders [3]. A substantial number of adolescents are considered at a high risk of developing eating disorders [4]. Furthermore, disordered eating can cause changes in adolescents’ cognitive functions and lead to depression [5] and an inclination for substance abuse [6].

The prevalence of disordered eating as measured by the Eating Attitudes Test-26 (EAT-26) varies across Europe and the United States [7]. A recent study conducted in Cyprus determined that 16.04% of adolescent boys and girls met the criteria for disordered eating [8]. A survey conducted among Greek adolescent boys and girls revealed a similar prevalence rate of 16.6% [9]. A study conducted among Slovakian adolescents discovered a disordered eating prevalence of 11.85% [10], and a survey conducted among Spanish adolescent girls revealed a prevalence of 10.1% [11]. A nationally representative survey of US high school participants discovered that 14.4% of adolescent girls and 3.8% of adolescent boys had disordered eating, as measured using the EAT-26 [12].

Recent studies have indicated that disordered eating has increased significantly in several Asian countries [13]. Studies using the EAT-26 have revealed disordered eating in 10.4% and 17.1% of two samples of adolescent Taiwanese girls [14, 15]. These percentages are similar to those of adolescent girls in South Korea (14.8%) [16]. In other Asian countries, the rates of disordered eating are noticeably higher. For example, 27.8% of Malaysian boys and girls [12], 26.6% of Hong Kongese girls [17], and 26.7% of Indian girls [18] have been revealed to exhibit eating disorders. These findings indicate that disordered eating among Asian adolescents, including those in Taiwan, is as prevalent or even more prevalent among Western countries.

Early models of disordered eating have indicated that its causes are complex and multifaceted [19]. The interpersonal model of binge eating proposed by Wilfley and Associates [20] suggested that negative responses from closely related people, family functioning, and interpersonal problems are associated with unhealthy weight control. The disordered eating model of Neumark-Sztainer and Associates [21] highlighted that socio-environmental factors (i.e., family–peer weight-related social norms and teasing behaviors, and family connectedness) could influence personal factors (i.e., bodyweight concerns, psychological well-being, and attitudes related to health and nutrition), leading to the development of unhealthy weight control behaviors (e.g., self-induced vomiting and other disordered eating behaviors). Tylka and Subich’s multidimensional model [22] emphasized social and cultural factors such as pressure for thinness and relational factors such as the social support of family and friends.

Numerous subsequent studies have confirmed that being teased about one’s weight by family members is related to adolescent binge eating behavior and disordered eating [23, 24]. A 5-year longitudinal study of 2516 adolescents in the United States revealed that adolescents with dieting peers were more likely to engage in extreme weight control behaviors [25]. In addition, psychological dysfunction resulting from peer bullying can lead to disordered eating behaviors in adolescents [26, 27]. A significant association between depression and disordered eating has been discovered in adolescents in Spain, China, and Malaysia [28–30]. A study conducted among American adolescents indicated that stress led to body dissatisfaction in boys, which led to dieting, but this dieting did not lead to bulimic symptoms [31].

Several studies on disordered eating among Taiwanese adolescents have revealed higher body dissatisfaction rates among girls than boys [32, 33]. In Taiwan, a survey of junior high school participants discovered that anxiety and weight-related self-stigmatization were strongly associated with emotional eating among girls. By contrast, body dissatisfaction was strongly related to emotional eating among boys [33]. Most studies on eating disorders in adolescents in Asia have involved only girls, and the predictors of disordered eating have typically been limited [32–34].

Additionally, the family environment also plays a role in developing disordered eating. Compared with children in traditional households (i.e., those with two biological or adoptive parents), adolescents in nontraditional family households (i.e., those with single parents, step-parents, or no parents) were more likely to display unhealthy eating habits such as skipping breakfast and lunch, eating fewer vegetables, and consuming more fast food; they also had less parental monitoring of meals [35]. Family household type has a more pronounced effect on the well-being of boys than that of girls in aspects such as anxiety, unhappiness, and self-esteem than girls [36]. The presence of grandparents or other adults such as aunts, uncles, and other relatives is associated with greater depressive symptoms [37]. Grandparents were reported to engage in significantly more maladaptive feeding practices such as using food to regulate emotions and restricting food [38]. A study on three-generation Chinese families in urban areas discovered that grandparents urge children to eat more meals and more significant portions at meals, and grandparents use food as an emotional tool [39].

Additionally, frequent parental weight talk was associated with adolescents using harmful weight control methods and poor psychological health [40]. For example, adolescents who perceived their father as authoritative tend to present the drive for thinness and body dissatisfaction [41]. Adolescents whose mothers and fathers engaged in weight-related conversations with them were more likely to diet, use unhealthy weight-control behaviors, and engage in binge eating [42]. These results emphasize the critical role of parenting style in eating disorder pathology. Parenting styles correlated with parents’ socioeconomic status, such as education, income, and occupation [43].

Several behavioral factors were also found associated with disordered eating behaviors and attitudes. Unhealthy exercise is a core symptom of eating disorders that predicts greater chronicity and risk of relapse [44]. Compulsive exercise to control weight/shape and compensate for recent food intake was associated with restrictive eating and binge eating [44]. Increased compulsive exercise is significantly associated with higher eating disordered symptoms [45], dietary restraint, weight, and shape concerns among clinically eating disordered patients [46]. Additionally, poor diet quality is also correlated with disordered eating. A study indicated that weight control behaviors were associated with greater odds of incident hyperlipidemia [47]. Adolescents and young adult current vegetarians were more likely to report binge eating with loss of control when compared to nonvegetarians [48].

The Eating Attitudes Test (EAT) is widely considered an indicator of eating disorder–related pathology. The EAT was developed in 1979 as a 40-item questionnaire later reduced to a 26-item instrument demonstrating high reliability [49]. Initially created for clinical samples, the EAT was also highly valid and reliable when used with nonclinical populations [50]. This study used the EAT-26 to measure disordered eating because the EAT-26 is used as the measurement tool in much of the literature on disordered eating among adolescents in diverse non-Western and Western contexts. The recent literature on disordered eating among Taiwanese adolescents has used the EAT-26 to assess disordered eating [15, 51–53]. This study’s use of the EAT-26 increased the relevance of its findings to the literature on disordered eating among adolescents [15, 28, 51–57].

However, studies that have used EAT 26 to investigate eating disorders in adolescents have investigated only females [12, 15, 16, 23, 34, 58]. Recent studies have revealed that eating disorders also exist among young males [59–61]. Men are comparable with women in terms of dietary restraint, driven exercise (i.e., exercising compulsively as a means of controlling one’s weight or body shape), and binge eating [59–61]. Because the EAT-26 was initially designed and tested exclusively with young (mean age: 21) female patients with anorexia nervosa [49], understanding how the EAT-26 functions in adolescent populations comprising both girls and boys are crucial.

### The present study

In summary, previous studies have revealed that disordered eating is determined by individual factors such as weight and body shape concerns [60, 61]; emotional distress [28, 29, 62]; by interpersonal factors, such as family influences [32, 33]; by peer relationships and negative responses from close ones [23, 49, 50]; and by macro-level environmental factors such as media and societal expectations [32]. This study adopted a multifaceted factor approach to examine gender-specific responses to multifaceted factors associated with disordered eating, including personal, behavioral, family, and school-related characteristics [23, 49, 50]. Based on previous reviews, we hypothesize that gender differences are significant at different levels of correlation with disordered eating, interpersonal and emotional influences are more critical on girls, and weight and body shape concerns on boys.

Furthermore, the study sample is collected from three schools in New Taipei City, Taiwan. New Taipei City has the largest population of immigrant marriages in Taiwan, and most of the immigrants come from Southeastern Asia. As indicated above, the prevalence rate of disordered eating in adolescents in Southeastern Asia seems relatively higher than other US or European, or Taiwan samples. We hypothesized that adolescents from immigrant families might be more likely to present disordered eating than adolescents from native families in Taiwan.

## Methods

### Survey development and measures

A cross-sectional study was conducted from March to June 2019 in New Taipei City, Taiwan. Three junior high schools were approached through the researchers’ contacts, and verbal approvals by the schools’ superintendents and teachers were obtained. Written informed consent forms were acquired from the participants and their legal guardians before they participated in this study. Each student completed a 20–25 min questionnaire in a classroom. A total of 1020 adolescents between the ages of 13 and 16 were surveyed from 42 classes in three junior high schools. The study was performed following the Declaration of Helsinki and was approved by Taiwan’s Research Ethics Committee (NTU NO. 201901HS030).

This study analyzed a sample of 729 adolescents (48.97% boys; mean age = 13.71 years; standard deviation = 0.93 years) from 40 classrooms in three junior high schools. A total of 157 participants (15.4%, 157 out of 1020 total participants) were excluded because of incomplete information regarding their parents’ informed consent. Among the 863 valid participants, a total of 134 participants (15.5%) were also excluded from the analysis due to missing values in the analytical variables or because the class response rate was below 50% (3 classes and 36 participants). Comparisons between the participants excluded (n = 134) and included (n = 729) in the study indicated no significant differences in demographic characteristics, such as gender, family and friend teasing, family and friend support, and depressive mood state, and EAT-26 scores (see details in Additional file 1: Table S1).

A structured, self-administrated questionnaire comprising the standardized EAT-26 in Chinese [53] and other questions was developed for data collection. A prior unpublished qualitative study was conducted in 2018. The researchers interviewed 30 junior high school participants in New Taipei City to understand the target sample’s eating behaviors and sugar-sweetened beverage consumption while drafting the initial questionnaire. Expert validation was used to assess the initial questionnaire. The questionnaire was then modified based on the opinions of three experts, including two junior high school teachers with administrative duties. Follow-up pilot testing was conducted with 37 participants to assess the psychometric properties of the questions, identify problems with missing data, determine the potential for bias, and ensure the consistency of responses [63]. Minor changes to the survey were implemented based on feedback from the pilot tests. The section containing sociometric network nomination questions was tested in a previous study conducted among 196 junior high school participants to ensure the reliability and validity of the questions [64].

Sociometric network nomination in a classroom environment has frequently been used to measure peer acceptance [65, 66]. The “close friend” nomination is a widely used measure of peer acceptance in the social network literature [67–70]. In Taiwan, participants are placed in a fixed group of classmates, and classroom-based friendships are typically a crucial segment of their broader friendship networks.

### Measures

*Eating Attitudes Test-26 (EAT-26)* The EAT-26 is a self-reported questionnaire that measures 26 items on a 6-point Likert scale with total scores ranging from 0 to 78. An EAT-26 score of 20 or higher indicated disordered eating [49]. Previous validations of the EAT-26 have reported good reliability, as indicated by coefficients ranging from 0.79 to 0.94 [50]. Cronbach’s alpha in this study’s sample was 0.86 (0.88 for males and 0.82 for females), indicating good internal consistency.

### Personal variables

*Gender* was coded as 0 = male and 1 = female.

*Perceived body weight* was measured by asking the participants to rate their body weight on a 4-point Likert scale as being underweight (1), normal (2), overweight (3), or obese (4).

*Depressive mood* was assessed using the Brief Symptom Rating Scale 5 (BSRS-5) [71]. This 5-item scale measures psychological symptoms such as feeling depressed or moody and has been demonstrated to have excellent validity and reliability in Taiwan. The score for each item ranges from 0 (*never*) to 4 (*very severe*), and the sum of these scores ranges from 0 to 20. A score of 10 and above indicates a moderate to severe depressive mood (coded as 1), which requires psychiatric attention. Cronbach’s alpha in this study’s sample was 0.85 (0.83 for males and 0.87 for females), indicating good internal consistency.

### Behavioral variables

*Unhealthy eating behaviors* were measured by asking participants whether they had consumed (1) sugar-sweetened beverages, (2) deep-fried food (e.g., fries and fried chicken), and (3) vegetables and fruit during the previous week (reversed coded). Participants answered these questions on a 5-point scale as follows: 0 = never, 1 = 1–3 times per week, 2 = 4–6 times per week, 3 = at least once per day, and 4 = every day. According to the WHO [72], healthy eating is conceptualized as a diet that helps protect against malnutrition in all its forms and non-communicable diseases. Healthy eating behaviors include but are not limited to reduced intake of saturated fats and trans-fats, salt, and free sugars, and increased consumption of fruits and vegetables. Therefore, we constructed an index of unhealthy eating behaviors by summing the scores of these three questions. The index scores range from 0 to 12.

*Regular exercise* was measured by asking participants how often they participated in physical activities that made them sweat or breathe more heavily than usual for at least 20 min; they replied with the following responses: never, once a month, less than four times per week, 4–6 days per week, or every day. According to the Taiwanese government’s official definition of regular exercise (https://www.gender.ey.gov.tw/gecdb/Stat_Statistics_DetailData.aspx?sn=lE9!VKlq9nql0ld4b!5R4w%40%40), participants aged 13–16 who exercise at least 90 min per week (e.g., 30-min exercise sessions, 3 days per week) are defined as engaging in regular exercise. Thus, in this study, participants who reported exercising 4–6 days per week (80 to 120 min) or every day (140 min) were defined as engaging in regular exercise.

### Family-related variables

*Perceived family support* was measured by student ratings of the extent of support provided by family members. Participants were asked four questions: whether they felt close to family members (emotional support); whether family members assisted when they were in need (instrumental support); whether family members provided them with advice when they were in need (informational support), and whether family members appreciated their thoughts and behaviors (appraisal support). Each question was scored on a 4-point Likert scale from 1 (strongly disagree) to 4 (strongly agree). Principle component analysis (PCA) revealed that one factor explained 67.14% of the total variance. Cronbach’s α for this scale was 0.83. The factor loadings are presented in Additional file 1: Table S2.

*Family weight-teasing* was measured by asking participants whether their family members made fun of or mocked them based on their weight or body shape. Participants responded with either a *Yes* or *No*.

*Family pressure to control weight* was measured by three items: (1) whether family members advised them to eat less; (2) whether family members suggested that they should refrain from eating snacks; and (3) whether family members suggested that they should control their weight. Participants replied to each item with a *Yes* or *No*. PCA revealed that one factor explained 58.26% of the total variance. Cronbach’s α was 0.64, and the three items were summed. The factor loadings are illustrated in Additional file 1: Table S2.

*Family type* for each respondent was categorized as those living with one parent, with both parents, or with grandparents. Adolescents living with two parents were classified as being part of a traditional family, and those living with only their fathers or mothers were categorized as part of a single-parent family. Those living with parents and grandparents were classified as extended family.

*An immigrant family* was defined as a family in which a father, a mother, or both parents held foreign nationality.

*The father’s level of education* was defined by whether the father had a college degree or above.

*The mother’s level of education* was defined by whether the mother had a college degree or above.

### School-related variables

*Low peer acceptance* was measured by asking each student to nominate up to five “close friends” in their class. Participants who received no nominations or only one “close friend” nomination were categorized as having low peer acceptance (coded 0 = *not low peer acceptance* or 1 = *low peer acceptance*).

*Perceived friend support* was measured by asking the participants four questions: whether they felt close with friends (emotional support); whether friends assisted when they were in need (instrumental support); whether friends provided them with advice when they were in need (informational support); and whether friends appreciated their thoughts and behaviors (appraisal support). Each question was scored on a 4-point Likert scale from 1 (*strongly disagree*) to 4 (*strongly agree*). PCA revealed that one factor explained 69.20% of the total variance, and Cronbach’s α for this scale was 0.85. The factor loadings are presented in Additional file 1: Table S2.

*Friend weight-teasing* was measured by asking participants whether their friends had made fun of or mocked them about their weight or body shape. Participants responded with a *Yes* or *No*.

*Peer pressure to control weight* was measured by three items: (1) whether friends had advised the respondents to eat less; (2) whether friends suggested that they should refrain from eating snacks; and (3) whether friends suggested that they should control their weight. Participants replied with a *Yes* or *No*. PCA revealed that one factor explained 62.37% of the total variance. The Cronbach’s α was 0.70. The factor loadings are presented in Additional file 1: Table S2.

### Data analysis

First, Chi-square and ANOVA F-tests were employed to examine gender differences in the multifaceted factors examined in this study and each of the EAT-26 items. Multiple logistic regressions were conducted, controlling for school cluster effects because of different prevalence rates of disordered eating across schools (Model 1). Stratification analysis was performed for each gender (Model 2 for females; Model 3 for males).

To control type I errors in the null hypothesis testing when conducting multiple comparisons, and we employed the false discovery rate (FDR) correction method formally described by Benjamini and Hochberg [73] (this method is also known as the BH correction procedure) to report the adjusted *P* values. This methodology is arguably a highly appropriate approach for distinguishing the few critical effects from the numerous effects tested [73]. The FDR is the *proportion* of incorrect rejections among all rejections of the null hypothesis. In this present study, given that the data is not from a national representative large-scale survey, we accepted a 10% false discovery rate instead of the general 0.25 (α/2, α = 0.5) [74].

The BH correction procedure proceeds as follows: first, put the individual *P* values in order, from smallest to largest. The smallest *P *value has a rank of *i* = 1, and then the next smallest has *i* = 2, etc. Compare each *P *value to its Benjamini–Hochberg critical value, (*i/m*)*Q*, where *i* is the rank, *m* is the total number of tests, and *Q* is the false discovery rate the researcher chooses. Generally, the largest *P *value that has *P* < (i/m)Q is significant, and *all* of the *P* values smaller than it are also significant [74]. The BH correction procedure has frequently been used in medical science and genome studies to conceptualize the rate of type I errors in the null hypothesis testing when conducting numerous comparisons. However, the use of FDR to report adjusted *P* values has not been common in public health and behavioral research.

## Results

### Sample characteristics

Approximately one-third of the participants (31.96%) perceived themselves as overweight or obese. A higher percentage of adolescent boys than girls perceived themselves as underweight (girls: 15.9%; boys: 25.5%, *P* < 0.001). Overall, the EAT-26 results revealed that 11.39% (n = 83) of the participants engaged in disordered eating attitudes or behaviors, and no gender difference was observed (girls: 11.56%; boys: 11.20%, *P* = 0.88). A total of 119 participants (16.32% of the total) reported having a moderate to severe depressive mood. More adolescent girls than boys reported having a moderate to severe depressive mood (19.89% vs. 12.60%, *P* = 0.01). The majority of the participants (64.60%, n = 471) lived in the traditional family structure, 16.73% (n = 122) lived with their extended families, and 18.66% (n = 136) lived with a single parent. A gender difference was observed: a higher percentage of adolescent girls than boys lived in single-parent families (22.04% vs. 15.12%, *P* = 0.05). Additionally, adolescent girls seemed to be under more family pressure than boys to control their weight (mean = 1.4 for girls and 1.19 for boys, *P* = 0.01). Approximately 15.91% (n = 116) of the participants were in the low peer acceptance category, and more adolescent boys than girls have low peer acceptance (19.32% vs.12.63%, *P* = 0.01). More details are shown in Table 1, and the Pearson’s correlations between all variables used are illustrated in Additional file 1: Table S3.

**Table 1:** Sample characteristics

	All (N = 729)		Females (N = 372, 51.03%)		Males (N = 357, 48.97%)		X^2^ (df)/F-test	*P* value
	N/Means	%/SD	N/Means	%/SD	N/Means	%/SD		
Age	13.83	0.99	13.83	1.00	13.84	0.97	0.03(1)	0.85
Perceivwd body image							11.73(2)	0.00
Normal weight	346	47.50	194	52.20	152	42.60		
Overweight/obese	233	31.96	119	32.00	114	31.90		
Underweight	150	20.60	59	15.90	91	25.50		
Disordered eating (EAT-26)								
Yes	83	11.40	43	11.60	40	11.20	0.02(1)	0.88
Friend weight-teasing								
Yes	184	25.20	103	27.70	81	22.70	2.41(1)	0.12
Family weight-teasing								
Yes	173	23.70	96	25.80	77	21.60	1.8(1)	0.10
Family pressure to control weight (0–3)	1.30	1.13	1.4	1.14	1.19	1.10	6.39(1)	0.01
Friend pressure to control weight	0.43	0.83	0.48	0.86	0.39	0.80	2.23(1)	0.14
Perceived family support (4–16)	12.30	2.62	12.35	2.73	12.26	2.50	0.22(1)	0.64
Perceived friend support (4–16)	12.42	2.44	12.57	2.38	12.27	2.50	2.62(1)	0.11
Low peer acceptance								
Yes	116	15.91	47	12.60	69	19.32	6.10(1)	0.01
Father with college degree or above								
Yes	229	31.40	109	29.30	120	33.60	1.57(1)	0.21
Mother with college degree or above								
Yes								
Immigrant family								
Yes	178	24.40	92	24.70	86	24.10	0.04(1)	0.84
Family type							5.97(2)	0.05
Traditional family	471	64.60	228	61.30	243	68.10		
Extended family	122	16.73	62	16.70	60	16.80		
Single-parent family	136	18.66	82	22.04	54	15.12		
Unhealthy eating behaviors (0–12)	6.22	2.41	6.12	2.37	6.32	2.46	6.98(1)	0.27
Regular execrise								
Yes	219	30.00	64	17.20	155	43.40	59.56(1)	0.00
Moderate to severe depressive mood								
Yes	119	16.30	74	19.89	45	16.32	7.08(1)	0.01

The top three items of the EAT-26 as scored by the total sample of participants (n = 729) were (1) being occupied with a desire to be thinner (41.29%, n = 301), (2) displaying self-control around food (40.05%, n = 292), and (3) being preoccupied with thoughts regarding their body fat (36.63%, n = 267), shown in Table 2. Gender was significantly associated with several specific items of the EAT-26. Adolescent boys were at disproportionately high risk for certain facets of disordered eating. For example, boys accounted for 58.06% (n = 18) of the 31 participants who had the impulse to vomit after meals, significantly greater than the 41.94% (n = 13) of girls in the group who reported vomiting after meals (*P* < 0.05). Boys accounted for 58.1% (n = 122) of the 210 participants reporting that they believe others perceive them to be underweight, compared with the 41.9% (n = 88) of girls in the group reporting this belief (*P* = 0.05). Boys also accounted for 61.02% (n = 36) of the 59 participants reporting that they like their stomachs to be empty, compared with the 38.98% (n = 23) of girls in the group who reported this preference (*P* = 0.05). However, girls accounted for two-thirds (66.7%, n = 174) of the 261 participants reporting the fear of being overweight, compared with the 33.3% (n = 87) of boys in this group (*P* < 0.001). Additionally, girls accounted for 64.45% (n = 194) of the 301 participants who reported a preoccupation with becoming thinner, compared with the 35.55% of boys (n = 107) reporting the same (*P* < 0.001). Girls also accounted for a higher percentage (65.9%, n = 176) of the 267 participants reporting a preoccupation with thoughts of body fat, compared with the 34.1% (n = 91) of boys reporting the same (*P* < 0.001).

**Table 2:** Disordered eating attitude, by gender (N = 729)

Items	All		Females^2^		Males		X^2^ (df)	*P* value
	N^1^	%	N	%	N	%		
1. I am terrified about being overweight	261	35.80	174	66.67	87	33.33	39.79(1)	0.00
2. I avoid eating when I am hungry	139	19.07	81	58.27	58	41.73	3.61(1)	0.06
3. I find myself preoccupied with food	121	16.60	70	57.85	51	42.15	2.70(1)	0.10
4. I have gone on eating binges where I feel that I may not be able to stop	65	8.92	27	41.54	38	58.46	2.57(1)	0.11
5. I cut my food into small pieces	170	23.32	95	55.88	75	44.12	2.09(1)	0.15
6. I aware of the calorie content of foods that I eat	97	13.31	45	46.39	52	53.61	0.96(1)	0.33
7. I particularly avoid food with a high carbohydrate content (i.e. bread, rice, potatoes, etc.)	71	9.74	35	49.30	36	50.70	0.10(1)	0.76
8. I feel that others would prefer if I ate more	207	28.40	96	46.38	111	53.62	2.50(1)	0.11
9. I vomit after I have eaten	11	1.51	4	36.36	7	63.64	0.96(1)	0.33
10. I feel extremely guilty after eating	47	6.45	25	53.19	22	46.81	0.09(1)	0.76
11. I am occupied with a desire to be thinner	301	41.29	194	64.45	107	35.55	36.97(1)	0.00
12. I think about burning up calories when I exercise	232	31.82	137	59.05	95	40.95	8.77(1)	0.00
13. I other people think that I am too thin	210	28.81	88	41.90	122	58.10	9.83(1)	0.00
14. I am preoccupied with the thought of having fat on my body	267	36.63	176	65.92	91	34.08	37.37(1)	0.00
15. I take longer than others to eat my meals	126	17.28	77	61.11	49	38.89	6.20(1)	0.01
16. I avoid foods with sugar in them	101	13.85	44	43.56	57	56.44	2.61(1)	0.11
17. I eat diet foods	28	3.84	11	39.29	17	60.71	1.61(1)	0.21
18. I feel that food controls my life	77	10.56	35	45.45	42	54.55	1.07(1)	0.30
19. I display self-control around food	292	40.05	140	47.95	152	52.05	1.85(1)	0.17
20. I feel that others pressure me to eat	95	13.03	44	46.32	51	53.68	0.97(1)	0.32
21. I give too much time and thought to food	80	10.97	39	48.75	41	51.25	0.19(1)	0.67
22. I feel uncomfortable after eating sweets	54	7.41	24	44.44	30	55.56	1.01(1)	0.31
23. I engage in dieting behavior	62	8.50	29	46.77	33	53.23	0.49(1)	0.48
24. I like my stomach to be empty	59	8.09	23	38.98	36	61.02	3.73(1)	0.05
25. I have the impulse to vomit after meals	31	4.25	13	41.94	18	58.06	8.38(1)	0.00
26. I enjoy trying new rich foods	549	75.31	297	54.10	252	45.90	1.07(1)	0.30

^1^N is the number of participants who reported they have this attitude or behaviors often, most of the time, always

^2^Row percentages were reported

The results from multiple regression analysis are shown in Table 3. The results demonstrated that participants who perceived themselves to be obese were more likely to engage in disordered eating than those who perceived themselves to be of normal weight, as indicated in Model 1 (adjusted odds ratio [AOR] = 1.33, *P* < 0.001; B–H critical *P* < 0.001). Additionally, the perception of being underweight had a protective effect against disordered eating relative to the perception of being of normal weight (AOR = 0.53, *P* < 0.001; B–H critical *P* < 0.001).

**Table 3:** Logistic regression on multifacted factors associated with disordered eating, by gender

	Model 1 (all)			B–H critical *P* value	Sig	Model 2 (females)			B–H critial *P* value	Sig	Model 3 (males)			B–H critial *P* value	Sig
	OR	95% CI	*P* value			OR	95% CI	*P* value			OR	95% CI	*P* value		
Personal variables															
Age	0.79	0.68–0.92	0.00	0.02	*	0.81	0.57–1.14	0.23	0.36		0.95	0.90–1.00	0.04	0.13	
Girls (ref: Boys)	0.96	0.66–1.41	0.84	0.88		NA					NA				
Perceived body weight (ref: Perceived normal weight)															
Perceived underweight	0.53	0.43–0.66	0.00	0.00	*	0.74	0.45–1.21	0.23	0.36		0.46	0.34–0.61	0.00	0.00	*
Perceived overweight	1.08	0.78–1.49	0.66	0.88		0.75	0.41–1.37	0.35	0.48		1.59	1.21–2.08	0.00	0.00	*
Perceived obese	1.33	1.12–1.59	0.00	0.00	*	0.40	0.14–1.12	0.08	0.17		1.88	0.70–5.10	0.21	0.36	
Moderate to severe depressive mood (ref: No)	3.79	2.23–6.47	0.00	0.00	*	6.74	3.66–12.42	0.00	0.00	*	1.64	0.49–5.51	0.42	0.50	
Behavioral variables															
Unhealthy eating behaviors	1.08	0.96–1.21	0.20	0.35		1.04	0.86–1.25	0.72	0.72		1.12	0.78–1.29	0.11	0.26	
Regular exercise (ref: No)	1.35	0.80–2.29	0.26	0.40		1.45	1.14–1.84	0.00	0.00	*	1.23	0.48–3.17	0.66	0.66	
Family-related variables															
Family weight-teasing (ref: No)	1.79	1.42–2.25	0.00	0.00	*	2.19	1.52–3.13	0.00	0.00	*	1.75	0.62–4.93	0.29	0.42	
Family pressure to control weight	1.23	0.70–2.15	0.47	0.63		1.30	0.73–2.30	0.38	0.48		1.24	0.69–2.24	0.47	0.49	
Perceived family support	1.01	0.81–1.26	0.93	0.93		0.94	0.77–1.13	0.50	0.59		1.08	0.84–1.38	0.55	0.58	
Mother with college degree and above (ref: No)	1.39	1.20–1.61	0.00	0.00	*	2.31	1.02–5.25	0.05	0.12		0.56	0.14–2.26	0.42	0.50	
Father with college degree and above (ref: No)	1.50	1.06–2.13	0.02	0.04	*	0.61	0.25–1.53	0.29	0.36		5.23	1.93–14.17	0.00	0.00	*
Immigrant family (ref: No)	2.14	2.07–2.21	0.00	0.00	*	2.05	1.66–2.54	0.00	0.00	*	2.80	2.13–3.68	0.00	0.00	*
Family type (ref: Traditional family)															
Single-parent family	1.49	0.70–3.15	0.30	0.43		0.79	0.23–2.78	0.72	0.72		2.98	0.68–13.06	0.15	0.30	
Extended family	0.97	0.74–1.28	0.83	0.88		1.92	1.64–2.24	0.00	0.00	*	0.45	0.19–1.06	0.07	0.19	
School-related variables															
Low peer acceptance in classroom (ref: No)	1.86	1.21–2.85	0.01	0.02	*	2.81	2.01–3.92	0.00	0.00	*	1.64	0.82–3.28	0.16	0.30	
Friend weight-teasing (ref: No)	1.09	0.54–2.17	0.81	0.88		0.81	0.28–2.31	0.69	0.72		2.03	0.55–7.53	0.29	0.42	
Friend pressure to control weight	1.48	0.93–2.33	0.10	0.20		1.63	1.05–2.52	0.02	0.05	*	1.33	0.72–2.45	0.36	0.53	
Perceived friend support	1.02	0.99–1.06	0.21	0.35		1.06	0.94–1.19	0.34	0.48		1.05	1.01–1.10	0.02	0.08	*
Constant	0.23	0.11–0.48	0.00			0.28	0.00–80.57	0.66			0.00	0.00–0.01			

B–H critial value is calcuated using the formula from Benjamini & Hochberg (1995)

*Indicates the B–H critical *P* value is significant at False Discovery Rate of 0.10

Participants who reported a moderate to severe depressive mood were 2.79 times more likely to engage in disordered eating (AOR = 3.79, *P* < 0.001; B–H critical *P* < 0.001) than other participants were. Older participants were less likely to engage in disordered eating than their younger peers were (AOR = 0.79, *P* < 0.001; B–H critical *P* = 0.02). Gender appeared no difference regarding the chance of engaging in disordered eating.

More participants (n = 184, 25.24%) reported being teased about their weight by peers than those who reported being teased about their weight by family members (n = 173, 23.73%). However, after adjustment for all the other variables, a strong association between weight-teasing by family and disordered eating (AOR = 1.79, *P* < 0.001; B–H critical *P* < 0.001) was discovered, whereas weight-teasing by peers was not found to have a significant association with disordered eating (*P* = 0.81). Additionally, participants with low peer acceptance were associated with disordered eating (AOR = 1.86, *P* < 0.01; B–H critical *P* < 0.01).

Regarding the family-related variables, participants from immigrant families had a higher likelihood of disordered eating. These participants were 2.14 times more likely to engage in disordered eating than participants with only Taiwanese parents (AOR = 2.14, *P* < 0.001; B–H critical *P* < 0.001). Participants with fathers or mothers with a college degree or above were 1.39 times and 1.50 times more likely, respectively (AOR = 1.39, *P* < 0.001; B–H critical *P* < 0.001; AOR = 1.50, *P* = 0.02; B–H critical *P* = 0.04), to engage in disordered eating than other participants were.

### Gender differences

When the results were disaggregated by gender, the factors associated with disordered eating differed significantly. Regarding personal and behavioral variables, the self-perception of body weight seems to be a more important factor for boys than girls. Adolescent boys who perceived themselves as overweight were more likely to engage in disordered eating than those who perceived themselves to be of normal weight (AOR = 1.59, *P* < 0.001; B–H critical *P* < 0.001). Additionally, the perception of being underweight had a protective effect against disordered eating relative to the perception of being of normal weight only for boys (AOR = 0.46, *P* < 0.001; B–H critical *P* < 0.001). The association between moderate to severe depressive mood and disordered eating was discovered to be significant only for girls (AOR for girls = 6.74, *P* < 0.001; B–H critical *P* < 0.001; AOR for boys = 1.64, *P* = 0.42; B–H critical *P* = 0.50). Adolescent girls who reported regular exercise were more likely than other participants were to engage in disordered eating (AOR = 1.45, *P* < 0.001; B–H critical *P* < 0.001), but no such association was discovered among boys.

Second, adolescent girls with a mother with a college education or above had a 2.31 times greater likelihood of engaging in disordered eating (AOR = 2.31, *P* = 0.05, B–H critical *P* = 0.12) than girls without a mother who had a college education or above. Similarly, having a father with a college degree or above was strongly associated with disordered eating among boys (AOR = 5.23, *P* < 0.00; B–H critical *P* < 0.01). However, after the BH-correction procedure, only fathers’ high level of education was significantly associated with disordered eating among boys. Girls whose families teased them about their weight had a 2.19 times greater likelihood of engaging in disordered eating than girls who were not teased about their weight by their families (AOR = 2.19, *P* < 0.001; B–H critical *P* < 0.001). However, no significant association between weight-teasing by family and disordered eating was discovered in boys. Girls who lived with their extended families (parents and grandparents) were 1.92 times more likely to exhibit disordered eating than girls who lived in traditional families (i.e., with two parents) (AOR = 1.92, *P* < 0.001; B–H critical* P* < 0.001). An immigrant family background was significantly associated with disordered eating among both boys (AOR = 2.80, *P* < 0.001; B–H critical *P* < 0.001) and girls (AOR = 2.05, *P* < 0.001; B–H critical *P* < 0.001). Finally, perceived family support had no significant influence on disordered eating among boys or girls.

Third, friend pressure to control one’s weight was determined to be significantly associated with disordered eating in girls (AOR = 1.63, *P* = 0.02; B–H critical *P* = 0.05), as was low acceptance by peers in the classroom (AOR = 2.81, *P* < 0.001; B–H critical *P* < 0.001). Notably, only perceived friend support was significantly associated with disordered eating among adolescent boys of those school-related variables. Adolescent boys who perceived peer social support were 5% (AOR = 1.05, *P* = 0.02; B–H critical *P* = 0.08) more likely to engage in disordered eating than those not. This result may suggest that more research is needed to understand the factors related to male-specific disordered eating patterns.

## Discussion

As identified using the EAT-26, the prevalence of disordered eating was within the range of rates reported by prior studies of adolescent Taiwanese women [14]. Numerous studies have reported that adolescent girls are at a higher risk of disordered eating than adolescent boys [3, 12, 16, 17, 32, 33, 58]. However, no significant gender differences were observed in our study sample's prevalence of disordered eating. This result is similar to that of a cross-sectional study conducted by Hautala et al. on 1036 adolescents that determined the prevalence of disordered eating to be only slightly higher among female participants than among male participants [75]. Recent studies have revealed that young men also engage in disordered eating [59–61]. Young men exhibit patterns similar to those of women in terms of dietary restraint, exercising compulsively as a means of controlling one’s weight or body shape, and binge eating [60]. Nevertheless, the multifaceted factors associated with male disordered eating patterns have not typically been recognized in the relevant literature.

Family and friend involvement in an adolescent’s weight control is associated with disordered eating by adolescents, and this relationship is particularly acute among girls. Adolescent girls who reported being teased by their family members about their weight were substantially more likely to exhibit disordered eating than those who were not teased. This finding is consistent with previous studies on non-Asian populations [32, 33]. Similarly, our result that low peer acceptance was associated with disordered eating only in girls was consistent with previous studies. However, differences in the definitions of low peer acceptance may limit the validity of such comparisons. For example, our study identified participants of low peer acceptance as those who were not nominated by at least one classmate as a “close friend.” By contrast, Eisenberg et al. assessed peer isolation using an established subscale [76].

As observed in previous studies, our findings did not indicate a significant association between friend teasing about weight and disordered eating among girls. However, the effects of friend teasing in our study could have been masked by the association of friend pressure to control one’s weight, significantly associated with disordered eating among adolescent girls. The high prevalence of family and peer weight-teasing (23.7% for family weight-teasing; 25.2% for peer weight-teasing) is a particular cause for concern and suggests the need for school-based educational interventions and family interventions to prevent disordered eating.

Several gender-specific differences related to specific facets of adolescent disordered eating attitudes were found. More girls than boys expressed a desire to become thinner and had serious concerns about being overweight. This finding was consistent with those of prior studies on disordered eating in Taiwan, which have indicated that a significantly higher percentage of adolescent girls than adolescent boys desired to be thinner [15] and that they had internalized the idea of a slim body [32]. However, in our study, more boys than girls reported an impulse to vomit after eating. Overall, we discovered that girls were more likely to appear preoccupied with fatness and a desire to be thinner. However, boys tended to engage in extreme dieting behaviors such as vomiting and retaining empty stomachs. A Spanish study reported a similar finding that adolescent boys exhibited more symptoms of bulimia than adolescent girls did [77]. The gender-specific correlates of disordered eating may reflect differences between cultures and populations, and these differences call for further investigation. More importantly, most of the family- and school-related variables were not significant correlates of disordered eating among boys. Thus, gender-based studies are warranted.

Our study also revealed a significant association between weight-teasing by family members and disordered eating among girls, but not among boys. Previous research on gender differences in family weight-teasing correlated with disordered eating has been mixed. A longitudinal study indicated a significant association between family weight-teasing and appearance anxiety in adolescent girls but not in boys [61]. However, a cohort study revealed a significant association between weight-related teasing by family members and binge eating among both adolescent boys and girls [78].

In addition, we discovered that girls had a 92% higher chance of engaging in disordered eating if they lived with their parents and grandparents. Still, this association was not revealed among boys. Recent studies have suggested that the intergenerational transmission of disordered eating occurs directly from mothers and grandmothers in families [79]. Our findings also indicate that the likelihood of engaging in disordered eating was 2.31 times higher among adolescent girls whose mothers had attained higher education and 5.23 times higher among boys whose fathers had achieved such education levels relative to their peers. The high expectations of highly educated parents, especially fathers, may exert substantial pressure on children as authoritative parental style correlates with high expectations of their children and may increase the drive for thinness and body dissatisfaction [33, 41]. In response to high parental expectations, disordered eating may be a form of stress-induced externalization undertaken by adolescents [80].

Adolescent boys and girls from immigrant families were more likely than their peers from nonimmigrant families to engage in disordered eating. Studies on children from immigrant families have indicated that poor socioeconomic circumstances and the stress associated with migration and acculturation frequently affect adolescents’ mental health [81]. However, limited research has been conducted on the effects of immigration and acculturation on stress-externalizing behaviors such as disordered eating. In our sample of predominantly Taiwanese individuals of Han Chinese descent, most immigrant families consisted of a mother from China, Vietnam, or Indonesia married to a Taiwanese man of Han Chinese ethnicity. In our data, the mothers of children from immigrant families were significantly less likely to attain higher education than mothers born in Taiwan (19.66% vs. 30.85%, *P* = 0.004). Yet, fathers from this type of family had a similar educational background (32.02% vs. 31.22%, *P* = 0.84). The disparity between the educational levels of fathers and mothers in immigrant families was significant (*P* = 0.005). In addition, the children of immigrant families reported less perceived family support than the children of Taiwanese-only parents did on measures such as whether family members provided them with advice or helped when needed (68.54% vs. 79.85%, *P* = 0.002) and whether family members appreciated their thoughts and behaviors (63.48% vs. 71.52%, *P* = 0.04) in the study sample. Whether the high prevalence of disordered eating is related to stress from the status-discordant immigrant family environment or to the social process of acculturation [54] warrants further research. Our findings corroborated a study in Spain that concluded that eating disorders were significantly associated with immigrant status among adolescent girls, but not boys [82]. When these results were analyzed according to the time that the respondents had lived in Spain, only female adolescent immigrants who had lived in Spain for less than 6 years were more significantly associated with eating disorders than male Spanish adolescents [82]. We did not ask study participants for the length of time that they or their immigrant parents had lived in Taiwan, although this factor may have influenced the discrepancy in the results. The substantial observed differences indicated that factors related to the family environment might result in adolescent girls having a higher chance of engaging in disordered eating than adolescent boys. Programs targeting adolescents’ health should thus consider family-based interventions.

Additionally, the school environment was determined to increase the likelihood of adolescent girls engaging in disordered eating behavior. As measured in a sociometric network, low peer acceptance was significantly associated with disordered eating among adolescent girls but not among boys. Girls generally tend to be subject to more peer pressure than boys to control their weight, including suggestions to eat less or refrain from snacking, which increases the probability of disordered eating. Helfert and Warschburger [83] revealed that German adolescent girls experienced more peer exclusion due to their appearance than adolescent boys did [83]. The relevant literature has demonstrated that adverse interpersonal relationships at home or school play an essential role in developing disordered eating among girls, which is also true in Taiwan.

Previous studies on gender differences in response to interpersonal influences during adolescence corroborate our study's results. These studies have revealed that significantly more adolescent girls than boys report interpersonal stress, including stress related to adverse events and problems involving family members, peers, and intimate relationships [84]. However, we also found that boys who perceived friend support were more likely to engage in disordered eating behavior. This result suggests that interpersonal influence on the development of disordered eating may have different mechanisms in girls and boys. Rudolph reviewed numerous relevant studies and concluded that significantly more girls than boys experience anxiety and depression [85]. Moreover, girls are more likely to blame themselves for relationship problems and are more concerned about negative peer evaluations [85]. Nevertheless, these findings underscore the importance of gender-sensitive educational interventions and family interventions in preventing adolescent disordered eating.

This study revealed that the perception of body weight is a more critical factor of engaging in disordered eating for boys than girls. Adolescent boys who perceived themselves as underweight were 54% less likely to engage in disordered eating than those who perceived themselves to be of normal weight. Similarly, boys who perceived themselves to be overweight have a 59% higher chance to engage in disordered eating than those perceived to be of normal weight. This finding support previous studies on disordered eating among Taiwanese adolescents that have discovered that anxiety and weight-related self-stigmatization were strongly associated with emotional eating among girls. By contrast, body dissatisfaction was strongly related to emotional eating among boys [33]. However, this finding conflicts with the study carried out by Haley, Hedberg, and Leman on 16,289 adolescents, which revealed that the self-perception of being underweight is associated with disordered eating behaviors such as prolonged fasting and vomiting [86]. However, the studies discussed previously were conducted among non-Asian populations. The differences between our findings and theirs suggest that differences exist between non-Asian people and Taiwanese adolescents, which warrants further examination.

Anxiety and depression are associated with disordered eating, but few prior studies have investigated the psychological factors specific to an Asian context [28, 54]. This study revealed that moderate to severe depressive mood is only significantly associated with disordered eating among girls. Prior research on the association between stress and disordered eating in Chinese women identified emotional restraint as a psychological factor specific to disordered eating in the Chinese context [28]. That study suggested that emotional restraint, common in the Chinese context, may lead to the psychosomatic expression of mental health conditions, including disordered eating [28].

Lastly, adolescent girls who reported regular exercise, defined as doing physical activities that made them sweat or breathe more heavily than usual for at least 20 min 4 to 7 days per week, were more likely to engage in disordered eating than their peers. About 30% of the participants (219 out of 729) reported regular exercise, and only 29.2% were girls (64 out of 219). However, the majority (82.7%) of the girls who reported regular exercise engaged in disordered eating (53 out of 64), as compared to 11.6% (18 out of 155) of the boys who reported regular exercise. A previous study revealed that unhealthy exercise is a core symptom of eating disorders that predicts greater chronicity and risk of relapse [44]. Compulsive exercise to control weight/shape and compensate for recent food intake was associated with restrictive eating, shape concerns, and binge eating [40–42]. This study did not employ the measurement of compulsive exercise, and the results can’t be compared with the findings in previous studies. Nevertheless, these findings underscore the importance of gender-sensitive compulsive exercise to control weight; a research area needs more attention.

## Limitations

The strengths and limitations of this study require consideration when interpreting its findings. This study’s key strength is its assessment of multifaceted factors, including personal, behavioral, family, and school variables potentially relevant to disordered eating among the genders in Taiwan; this led to the discovery of novel gender differences regarding the correlates of disordered eating. However, the limitations of our study should also be acknowledged. First, the sample we examined was not a random national sample, limiting the generalizability of the results. Second, our study focused on Taiwanese adolescents in schools, and comparisons with other populations should be performed with care. Third, our findings are subject to recall bias and temporal ambiguity due to the nature of cross-sectional studies. Fourth, the measures adopted in this study were brief and based on self-report. Fifth, the name generator used to solicit the names of “close friends” from the participants without defining what “close friend” means may create confusion.

This study’s findings suggest a need to explore other gender differences in an extensive and comprehensive population-based study. In addition, alternative measures of disordered eating can be examined. For example, the Children’s Eating Attitudes Test has been used in some studies on disordered eating among adolescents [77, 87]. The Eating Disorder Examination Questionnaire is another commonly used tool to measure disordered eating in Western contexts [88–90]. Moreover, the Eating Disorders Inventory-2 has been applied to measure disordered eating in Western and Southeast Asian contexts [56, 57]. Further research should be undertaken to compare these measures in different cultural contexts and populations.

## Conclusion

Our findings suggest that future interventional studies should be designed to decrease the effects of adverse weight-related norms in schools and families. Gender-sensitive and interpersonal stress prevention programs are necessary to reduce the prevalence of body dissatisfaction, binge eating, and extreme weight control behaviors among adolescents.

This study’s findings underscore the importance of various promotional and protective factors—including personal characteristics such as the perception of one’s body weight and depressive mood, family and school environment factors, and behavioral factors—for disordered eating among adolescents. The use of a standardized disordered eating scale (i.e., the EAT-26) allowed for international and domestic comparisons regarding the prevalence of disordered eating. Our study provides a direction for future studies examining this population, and it may also facilitate the development of interventions to reduce the prevalence of disordered eating among adolescents.

## Supplementary Information

**Additional file 1.** **Table S1.** Differences between included and excluded participants (N = 863). **Table S2.** Factor loadings of family and friend pressure to control weight, perceived family support, perceived friend support and depressive mood (N = 729). **Table S3.** Correlation among variables in this study (N = 729).

## Data Availability

Data is available upon request.

## Abbreviations

EAT-26: Eating Attitudes Test-26
AOR: Adjusted odds ratios
BSRS-5: Brief Symptom Rating Scale 5
PCA: Principal component analysis
