# Supplementary material for: Gender-specific responses to multifaceted factors associated with disordered eating among adolescents of 7th to 9th grade
Source: J Eat Disord. 2022 Jan 10;10:5. doi: 10.1186/s40337-021-00524-3 (PMC8751146; doi:10.1186/s40337-021-00524-3)
Supplement: Supplementary file 1 — Additional file 1. Table S1. Differences between included and excluded participants (N = 863). Table S2. Factor loadings of family and friend pressure to control weight, perceived family support, perceived friend support and depressive mood (N = 729). Table S3. Correlation among variables in this study (N = 729). [file 40337_2021_524_MOESM1_ESM.docx]

| **Table S1 Differences between included and excluded participants (N=863)** | | | |  |  |  |
| --- | --- | --- | --- | --- | --- | --- |
|  | Included (N=729) | | Excluded (N=134) | | X^2^ (df) | P-value |
|  | N/Means | *%/*SD | N/Means | %/SD |  |  |
| Disordered eating (EAT-26) |  |  |  |  |  |  |
| Yes | 84 | 11.50 | 16 | 11.9 | 0.02(1) | 0.89 |
| Famiy weight-teasing |  |  |  |  |  |  |
| Yes | 171 | 23.70 | 28 | 21.1 | 0.45(1) | 0.50 |
| Peer weight-teasing |  |  |  |  |  |  |
| Yes | 184 | 25.40 | 33 | 24.8 | 0.02(1) | 0.88 |
| Immigrant family |  |  |  |  |  |  |
| Yes | 183 | 26.90 | 29 | 23.4 | 0.67(1) | 0.41 |
| Low peer acceptance in classrooms |  |  |  |  |  |  |
| Yes | 109 | 16.20 | 7 | 12.3 | 0.61(1) | 0.44 |
| Perceived family support (4-16) | 12.29 | 2.67 | 12.69 | 2.49 | 18.27(1) | 0.11 |
| Perceived peerl support (4-16) | 12.37 | 2.53 | 12.82 | 2.42 | 22.63(1) | 0.06 |
| Family norms for control weight (0-3) | 1.31 | 1.12 | 1.38 | 1.16 | 0.49(1) | 0.53 |
| Peer norms for control weight (0-3) | 0.46 | 0.86 | 0.42 | 0.77 | 0.14(1) | 0.66 |
| Depressive mood |  |  |  |  |  |  |
| Yes | 114 | 17.20 | 5 | 8.9 | 2.56(1) | 0.11 |

| **Table S2 Factor loadings of family and friend pressure to control weight, perceived family support, perceived friend support and depressive mood (N=729)** | | | |
| --- | --- | --- | --- |
|  | **Factor loadings** | **Cronbach α** | **Variance explained** |
| **Family pressure to control weight** |  | 0.64 | 58.26% |
| Whether family members had advised to eat less | 0.82 |  |  |
| Whether family members had suggested ro refrain from eating snacks | 0.67 |  |  |
| Whether family members had suggested to control their weight | 0.76 |  |  |
| **Friend pressure to control weight** |  | 0.70 | 62.37% |
| Whether friends advised to eat less | 0.81 |  |  |
| Whether friends suggested yo refrain from eating snacks | 0.80 |  |  |
| Whether friends suggested to control their weight | 0.76 |  |  |
| **Perceived family support** |  | 0.83 | 67.14% |
| Whether felt close with family members (emotional support) | 0.82 |  |  |
| Whether family members assisted when you were in need (instrumental support) | 0.86 |  |  |
| Whether family members gave advice when you were in need (informational support) | 0.82 |  |  |
| whether family members appreciated your thoughts and behaviors (appraisal support) | 0.78 |  |  |
| **Perceived friend support** |  | 0.85 | 69.20% |
| Whether felt close to friends (emotional support) | 0.81 |  |  |
| Whether friends assisted when you were in need (instrumental support) | 0.87 |  |  |
| Whether friends gave advice when in need (informational support) | 0.85 |  |  |
| Whether friends appreciated your thoughts and behaviors (appraisal support) | 0.79 |  |  |

**Table S3. Correlation among variables in this study (N=729)**

|  | | Family weigh-teasing | Friend weigh-teasing | Age | Disordered eating | Low peer acceptance | Immigrant family | Regular exercise | Perceived underweight | Perceived obese | Perceived overweight | woman | Friend pressure to control weight | Family pressure to control weight | Family support | Friend support | Unhealthy eating | |
| --- | --- | --- | --- | --- | --- | --- | --- | --- | --- | --- | --- | --- | --- | --- | --- | --- | --- | --- |
| Family weight-teasing | Pearson’s r | 1 |  |  |  |  |  |  |  |  |  |  |  |  |  |  |  | |
|  | P-value |  |  |  |  |  |  |  |  |  |  |  |  |  |  |  |  | |
| Friend weight-teasing | Pearson’s r | .418^**^ | 1 |  |  |  |  |  |  |  |  |  |  |  |  |  |  | |
|  | P-value | .000 |  |  |  |  |  |  |  |  |  |  |  |  |  |  |  | |
| Age | Pearson’s r | -.032 | .039 | 1 |  |  |  |  |  |  |  |  |  |  |  |  |  | |
|  | P-value | .386 | .297 |  |  |  |  |  |  |  |  |  |  |  |  |  |  | |
| Disordered eating | Pearson’s r | .196^**^ | .179^**^ | -.053 | 1 |  |  |  |  |  |  |  |  |  |  |  |  | |
|  | P-value | .000 | .000 | .156 |  |  |  |  |  |  |  |  |  |  |  |  |  | |
| Low peer acceptance | Pearson’s r | .031 | .032 | -.074^*^ | .092^*^ | 1 |  |  |  |  |  |  |  |  |  |  |  | |
|  | P-value | .409 | .386 | .046 | .013 |  |  |  |  |  |  |  |  |  |  |  |  | |
| Immigrant family | Pearson’s r | .013 | .037 | .016 | .098^**^ | -.012 | 1 |  |  |  |  |  |  |  |  |  |  | |
|  | P-value | .722 | .315 | .660 | .008 | .755 |  |  |  |  |  |  |  |  |  |  |  | |
| Regular exercise | Pearson’s r | .011 | -.002 | -.031 | .038 | .026 | -.045 | 1 |  |  |  |  |  |  |  |  |  | |
|  | P-value | .773 | .959 | .410 | .302 | .487 | .224 |  |  |  |  |  |  |  |  |  |  | |
| Perceived underweight | Pearson’s r | -.097^**^ | -.120^**^ | .011 | -.101^**^ | -.007 | .017 | -.007 | 1 |  |  |  |  |  |  |  |  | |
|  | P-value | .009 | .001 | .767 | .006 | .848 | .652 | .846 |  |  |  |  |  |  |  |  |  | |
| Perceived obese | Pearson’s r | .102^**^ | .254^**^ | .024 | .105^**^ | .027 | .025 | -.013 | -.144^**^ | 1 |  |  |  |  |  |  |  | |
|  | P-value | .006 | .000 | .518 | .005 | .467 | .504 | .736 | .000 |  |  |  |  |  |  |  |  | |
| Perceived overweight | Pearson’s r | .250^**^ | .196^**^ | -.004 | .101^**^ | .079^*^ | .000 | -.076^*^ | -.279^**^ | -.164^**^ | 1 |  |  |  |  |  |  | |
|  | P-value | .000 | .000 | .909 | .007 | .033 | .996 | .040 | .000 | .000 |  |  |  |  |  |  |  | |
| Woman | Pearson’s r | .050 | .058 | -.006 | .006 | -.091^*^ | .007 | -.286^***^ | -.103^**^ | -.052 | .033 | 1 |  |  |  |  |  | |
|  | P-value | .179 | .121 | .867 | .880 | .013 | .841 | .000 | .005 | .161 | .370 |  |  |  |  |  |  | |
| Friend pressure to control weight | Pearson’s r | .198^**^ | .408^**^ | .012 | .208^**^ | .017 | .015 | .000 | -.112^**^ | .199^**^ | .103^**^ | .055 | 1 |  |  |  |  | |
|  | P-value | .000 | .000 | .741 | .000 | .651 | .691 | .995 | .002 | .000 | .005 | .136 |  |  |  |  |  | |
| Family pressure to control weight | Pearson’s r | .261^**^ | .275^**^ | -.093^*^ | .146^**^ | -.016 | -.041 | -.055 | -.187^**^ | .213^**^ | .300^**^ | .093^*^ | .310^**^ | 1 |  |  |  | |
|  | P-value | .000 | .000 | .012 | .000 | .663 | .268 | .136 | .000 | .000 | .000 | .012 | .000 |  |  |  |  | |
| Family support | Pearson’s r | -.136^**^ | -.101^**^ | -.123^**^ | -.042 | -.051 | -.075^*^ | -.005 | -.007 | -.034 | -.055 | .018 | -.017 | .123^**^ | 1 |  |  | |
|  | P-value | .000 | .006 | .001 | .261 | .173 | .044 | .885 | .844 | .361 | .141 | .637 | .656 | .001 |  |  |  | |
| Friend support | Pearson’s r | -.078^*^ | -.105^**^ | -.008 | -.032 | -.208^**^ | -.006 | .133^***^ | .003 | -.051 | -.090^*^ | .060 | -.014 | -.078^*^ | .361^**^ | 1 |  | |
|  | P-value | .034 | .005 | .819 | .386 | .000 | .875 | .000 | .927 | .173 | .015 | .106 | .703 | .034 | .000 |  |  | |
| Unhealthy eating | Pearson’s r | .066 | .051 | .072 | .071 | .016 | .056 | -.004 | .070 | .096^**^ | .016 | -.041 | .066 | -.057 | -.116^**^ | .020 | 1 | |
|  | P-value | .077 | .172 | .051 | .054 | .658 | .134 | .918 | .060 | .009 | .657 | .274 | .077 | .122 | .002 | .584 |  | |
| **. Correlation at p<0.01. | | | | | | | | | | | | | | | | | |  |
| *. Correlation at p<0.05. | | | | | | | | | | | | | | | | | |  |
